# Divergent Effects of the N-Methyl-D-Aspartate Receptor Antagonist Kynurenic Acid and the Synthetic Analog SZR-72 on Microcirculatory and Mitochondrial Dysfunction in Experimental Sepsis

**DOI:** 10.3389/fmed.2020.566582

**Published:** 2020-11-27

**Authors:** László Juhász, Attila Rutai, Roland Fejes, Szabolcs P. Tallósy, Marietta Z. Poles, Andrea Szabó, István Szatmári, Ferenc Fülöp, László Vécsei, Mihály Boros, József Kaszaki

**Affiliations:** ^1^Faculty of Medicine, Institute of Surgical Research, University of Szeged, Szeged, Hungary; ^2^Research Group for Stereochemistry, Institute of Pharmaceutical Chemistry, Hungarian Academy of Sciences, University of Szeged, Szeged, Hungary; ^3^Department of Neurology, Interdisciplinary Excellence Centre, Faculty of Medicine, University of Szeged, Szeged, Hungary; ^4^Hungarian Academy of Sciences (MTA)-University of Szeged (SZTE), Neuroscience Research Group, Szeged, Hungary

**Keywords:** polymicrobial sepsis, kynurenic acid, N-methyl-D-aspartate receptor antagonist, microcirculation, mitochondrial respiration, organ dysfunction

## Abstract

**Introduction:** Sepsis is a dysregulated host response to infection with macro- and microhemodynamic deterioration. Kynurenic acid (KYNA) is a metabolite of the kynurenine pathway of tryptophan catabolism with pleiotropic cell-protective effects under pro-inflammatory conditions. Our aim was to investigate whether exogenously administered KYNA or the synthetic analog SZR-72 affects the microcirculation and mitochondrial function in a clinically relevant rodent model of intraabdominal sepsis.

**Methods:** Male Sprague–Dawley rats (*n* = 8/group) were subjected to fecal peritonitis (0.6 g kg^−1^ feces ip) or a sham operation. Septic animals were treated with sterile saline or received ip KYNA or SZR-72 (160 μmol kg^−1^ each) 16 and 22 h after induction. Invasive monitoring was performed on anesthetized animals to evaluate respiratory, cardiovascular, renal, hepatic and metabolic dysfunctions (PaO_2_/FiO_2_ ratio, mean arterial pressure, urea, AST/ALT ratio and lactate levels, respectively) based on the Rat Organ Failure Assessment (ROFA) score. The ratio of perfused vessels (PPV) of the ileal serosa was quantified with the intravital imaging technique. Complex I- and II-linked (CI; CII) oxidative phosphorylation capacities (OXPHOS) and mitochondrial membrane potential (ΔΨmt) were evaluated by High-Resolution FluoRespirometry (O2k, Oroboros, Austria) in liver biopsies. Plasma endothelin-1 (ET-1), IL-6, intestinal nitrotyrosine (NT) and xanthine oxidoreductase (XOR) activities were measured as inflammatory markers.

**Results:** Sepsis was characterized by an increased ROFA score (5.3 ± 1.3 vs. 1.3 ± 0.7), increased ET-1, IL-6, NT and XOR levels, and decreased serosal PPV (65 ± 12% vs. 87 ± 7%), ΔΨmt and CI–CII-linked OXPHOS (73 ± 16 vs. 158 ± 14, and 189 ± 67 vs. 328 ± 81, respectively) as compared to controls. Both KYNA and SZR-72 reduced systemic inflammatory activation; KYNA treatment decreased serosal perfusion heterogeneity, restored PPV (85 ± 11%) and complex II-linked OXPHOS (307 ± 38), whereas SZR-72 improved both CI- and CII-linked OXPHOS (CI: 117 ± 18; CII: 445 ± 107) without effects on PPV 24 h after sepsis induction.

**Conclusion:** Treatment with SZR-72 directly modulates mitochondrial respiration, leading to improved conversion of ADP to ATP, while administration of KYNA restores microcirculatory dysfunction. The results suggest that microcirculatory and mitochondrial resuscitation with KYNA or the synthetic analog SZR-72 might be an appropriate supportive tool in sepsis therapy.

## Introduction

Treatment of sepsis-induced multi-organ failure (MOF) is one of the most challenging tasks in intensive care therapy (1). According to current knowledge, the key problem in sepsis is the oxygen extraction deficit, which can originate from either insufficient oxygen delivery to the cells or inability of the cells to utilize oxygen. The poorly functioning microvasculature reduces delivery of oxygen to the tissue. In addition, as the mitochondrial electron transport system (ETS) is insufficient, it is unable to use oxygen efficiently; the switch to anaerobic pathways thus causes an energy deficit and eventual cell death (2, 3). These processes are intimately linked and finally lead to microcirculatory and mitochondrial distress syndrome (MMDS), which is believed to mediate end-organ damage (4). Therefore, the cornerstone of current organ-protective therapies is to prevent and treat oxygen debt globally by increasing oxygen uptake and transport, providing an adequate supply to meet subcellular oxygen demand (5). However, currently used respiratory- and circulatory-supportive modalities cannot always improve sepsis-induced alterations at the later stages (6).

Given this background, the major goal of our study was to find a novel, clinically applicable maneuver for microcirculatory recruitment and mitochondrial resuscitation to minimize the energy deficit of the organs. The metabolites and end-products of the tryptophan–l-kynurenine pathway have already been implicated in several ischemic and inflammatory disorders in the central nervous system (7). This pathway generates excitotoxic, N-methyl-d-aspartate receptor (NMDA-R) agonist quinolinic acid and the glutamate receptor antagonist kynurenic acid (KYNA). Interestingly, sepsis-induced tissue hypoxia is also associated with the activation of NMDA-R, which can lead to glutamate excitotoxicity and oxidative/nitrosative stress-mediated cell damage (8). Further, elevated plasma KYNA levels have been reported in association with pro-inflammatory cytokines and increased lactate concentrations in septic shock patients (9).

Excessive NMDA-R activation has been documented in various experimental models of inflammatory bowel diseases (10). Apart from neurons, the expression of receptor subunits was confirmed in peripheral organs, including the heart, small intestine, pancreas, and liver (11), where the activation of cellular NMDA-Rs may initiate oxidative stress, mitochondrial dysfunction, and apoptosis through calcium (Ca^2+^)- and reactive oxygen species (ROS)-mediated pathways (12).

Based on these findings, we hypothesized that KYNA via the inhibition of NMDA-R or other mechanisms might be a therapeutic tool to reduce microcirculatory and mitochondrial disturbances in sepsis. KYNA has a high affinity for the glycine co-agonist site on NMDA-R, binds to orphan G protein-coupled receptor GPR35 and aryl hydrocarbon receptor (13), and therefore takes part in the modulation of glutamatergic neurotransmission and alleviates adaptive immune response. Despite these properties and pleiotropic effects, its role in the regulation of the circulatory system is still unclear, and KYNA is considered a non-receptor-specific molecule (14). To address this issue, we set out to characterize and compare the microcirculatory and mitochondrial effects of KYNA and its blood–brain barrier (BBB)-permeable synthetic analog, SZR-72 (15, 16), on sepsis-induced microcirculatory and mitochondrial abnormalities and organ failure in a clinically-relevant rodent model of intra-abdominal sepsis.

## Materials and Methods

### Animals

Male Sprague–Dawley rats (*n* = 32; 350 ± 30 g) were used. The animals were housed in plastic cages (21–23°C) with a 12/12 h dark/light cycle and access to standard rodent food and water *ad libitum*. The experiments were performed in accordance with the National Institutes of Health guidelines on the handling and care of experimental animals and the EU Directive 2010/63 for the protection of animals used for scientific purposes (approval number V/175/2018).

### Sepsis Induction

Polymicrobial sepsis was induced with 3 ml intraperitoneally (ip)-administered fecal inoculum as described before (17). Briefly, fresh feces was collected from different animals and suspended in physiological saline. The concentration of the microorganisms in the suspension was determined before injection in 0.6 g kg^−1^ final doses.

### Experimental Protocol

The animals were randomly divided into sham-operated (*n* = 8) and sepsis (*n* = 24) groups. Septic animals were subjected to fecal peritonitis (0.6 g kg^−1^ feces ip) or a sham operation (sterile saline ip). After 6 and 16 h from sepsis induction, all the animals received balanced crystalloid solution (Ringerfundin, 1.5 ml kg^−1^; B. Braun, Melsungen, Germany) and analgesics (Buprenorphine, 15 μg kg^−1^; Richter Pharma, Hungary) subcutaneously. Septic animals were divided further into KYNA- (Sigma-Aldrich Inc., St. Louis, MO, USA; 160 μmol kg^−1^ ip; *n* = 8) or SZR-72- [2-(2-N,N-dimethylaminoethyl-amine-1-carbonyl)-1H-quinolin-4-one hydrochloride, synthesized by the Institute of Pharmaceutical Chemistry, University of Szeged, Hungary; 160 μmol kg^−1^ ip; *n* = 8] treated and vehicle-treated control (saline ip; *n* = 8) groups. Treatments were performed in two steps (80 μmol kg^−1^; in 1 ml kg^−1^ saline each) 16 and 22 h after sepsis induction.

To follow the progression of sepsis, the health status of the animals was monitored with a standardized well-being scoring system originally described for mice (18, 19). At the 22nd hour of the study, all the animals were anesthetized (ip ketamine 45.7 mg kg^−1^ and xylazine 9.12 mg kg^−1^) and placed on a heating pad to maintain normal core body temperature (37°C). A tracheostomy was performed to facilitate spontaneous breathing, and the right jugular vein was cannulated for fluid resuscitation (Ringerfundin, 10 ml^−1^ kg^−1^ h^−1^; B. Braun, Melsungen, Germany) and for the maintenance of continuous anesthesia (ketamine 12 mg kg^−1^ h^−1^, xylazine 2.4 mg kg^−1^ h^−1^, and diazepam 0.576 mg kg^−1^ h^−1^ iv). The left carotid artery was cannulated to monitor heart rate (HR) and mean arterial pressure (MAP) (SPEL Advanced Cardiosys 1.4; Experimetria Ltd., Budapest, Hungary).

**Figure 1:**
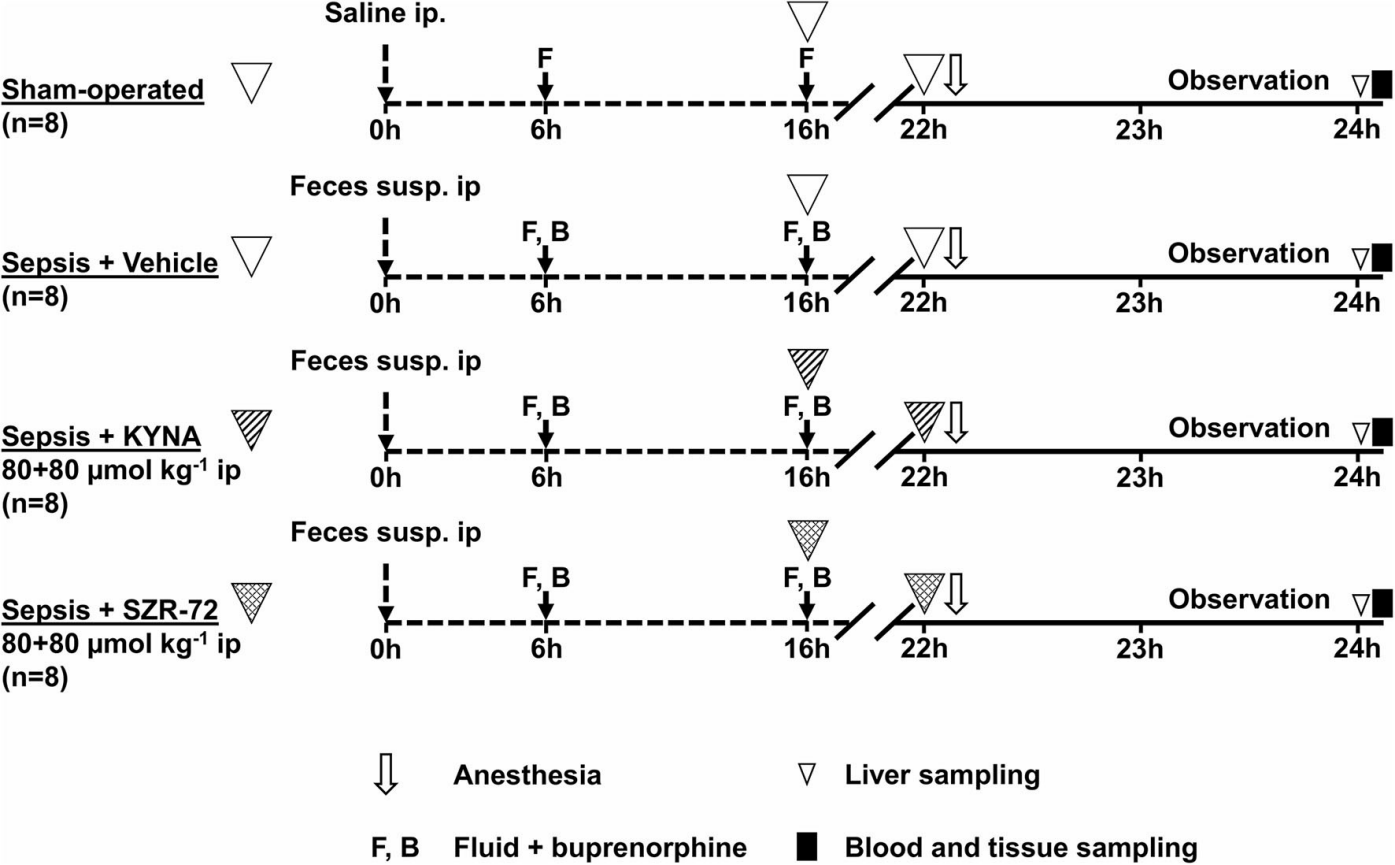
The experimental scheme. The animals were randomly assigned into the sham-operated and sepsis groups. Sixteen hours after sepsis induction, animals were randomly divided into three subgroups: treated with vehicle, KYNA (80 + 80 μmol kg^−1^ ip), or SZR-72 (80 + 80 μmol kg^−1^ ip), respectively.

After surgery and a 30-min stabilization, MAP and HR monitoring was performed every 15 min for 60 min. Arterial and venous blood gas analyses (Cobas b123; Roche Ltd., Basel, Switzerland) were performed at the 0th and 60th min of the monitoring period. Simplified oxygen extraction (O_2_ER) was calculated from arterial and venous oxygen saturation based on a standard formula (SaO_2_ – SvO_2_)/SaO_2_). The degree of lung injury was determined by using the arterial partial pressure of oxygen to fractional inspired oxygen (PaO_2_/FiO_2_) ratio. After the 60-min hemodynamic monitoring period, a median laparotomy was performed to observe the microcirculation of the ileal serosa (see below). Thereafter, a liver tissue biopsy was taken to evaluate mitochondrial respiratory functions. Samples from the terminal ileum were harvested, followed by blood sampling from the inferior vena cava for biochemical measurements (see below). After tissue samplings, animals were sacrificed under deep anesthesia (Figure 1).

### Measurements of Metabolic, Inflammatory, and Organ Function-Related Markers

Whole blood lactate levels were measured from venous blood samples (Accutrend Plus Kit; Roche Diagnostics Ltd., Rotkreuz, Switzerland) to determine metabolic imbalance. After the liver sampling, blood samples were collected from the inferior caval vein into precooled, EDTA-containing tubes (1 mg ml^−1^), centrifuged (1,200*g* at 4°C for 10 min), and stored at −70°C. Plasma endothelin-1 (ET-1) and interleukin-6 (IL-6) levels were determined with standard ELISA kits (Cusabio Biotechnology Ltd., Wuhan, China and Biomedica Ltd., Vienna, Austria, respectively) following the manufacturer's instructions. Kidney injury was determined from plasma urea level, whereas liver dysfunction was assessed by measuring plasma alanine aminotransferase (ALT) and aspartate aminotransferase (AST) levels, using a Roche/Hitachi 917 analyzer (F. Hoffmann-La Roche AG, Switzerland). The De Ritis ratio (AST/ALT ratio) was calculated as a marker of hepatocellular damage. All analyses were performed on coded samples in a blinded fashion.

### Evaluation of Ileal Xanthine Oxidoreductase Activity and Nitrotyrosine Levels

The same snap frozen tissues were used for these measurements. Ileum biopsies kept on ice were homogenized with a rotor stator homogenizer in a phosphate buffer (pH 7.4) containing 50 mmol l^−1^ Tris–HCl, 0.1 mmol l^−1^ EDTA, 0.5 mmol l^−1^ dithiothreitol, 1 mmol l^−1^ phenylmethylsulfonyl fluoride, 10 μg ml^−1^ soybean trypsin inhibitor, and 10 μg ml^−1^ leupeptin. Homogenates were then centrifuged at 4°C for 10 min at 15,000*g*, and the supernatant was then divided into one ultra-filtered part and one unfiltered one, which were used for xanthine oxidoreductase (XOR) activity and nitrotyrosine determination, respectively.

XOR activity was measured in the ultra-filtered supernatant (Amicon Ultra-0.5 Centrifugal Filter) with a fluorometric kinetic assay on the basis of the conversion of pterin to isoxanthopterin in the presence (total XOR) or absence (xanthine oxidase activity) of the electron acceptor methylene blue (20). XOR activity was calculated and expressed in μmol min^−1^ mg protein^−1^.

Free nitrotyrosine as a marker of peroxynitrite generation was measured from unfiltered supernatant by enzyme-linked immunosorbent assay (Cayman Chemical, Ann Arbor, MI, USA). The supernatants were incubated overnight with anti-nitrotyrosine rabbit IgG and nitrotyrosine acetylcholinesterase tracer in precoated (mouse anti-rabbit IgG) microplates, followed by development with Ellman's reagent, and measured spectrophotometrically at 405 and 420 nm. Tissue nitrotyrosine content was calculated in ng mg^−1^ protein. Protein content was assessed by Lowry's method.

### Rat Organ Failure Score Assessment

The severity of organ failure was determined by using a scoring system adapted for rats (Rat Organ Failure Assessment—ROFA) considering the principles of the Sepsis-3 international consensus (Table 1). ROFA components were scored between 0 and 4 based on threshold values of different parameters (21). The cardiovascular (MAP values) and respiratory components (the PaO_2_/FiO_2_ ratio) of ROFA were determined from readings of hemodynamic and blood gas monitoring, respectively. Sepsis-induced liver damage was determined by calculating the AST/ALT ratio (De Ritis ratio) (22). Renal dysfunction was characterized by determining plasma urea levels. The ROFA scoring system was supplemented by scoring the degree of blood lactate level (indicative of metabolic disturbances caused by tissue hypoxia). The ROFA values were calculated by summing up the scores in each element of the scoring system. Septic status was defined as a ROFA score above 2.

**Table 1:** Threshold values of the Rat Organ Failure Assessment (ROFA) scoring system for the individual organ dysfunction parameters.

		**ROFA score**				
**Organ dysfunction**	**Parameters**	**0**	**1**	**2**	**3**	**4**
Respiratory system	PaO_2_/FiO_2_ ratio	≥400	<400	<300	<200	<100
Cardiovascular system	MAP (mmHg)	≥75	65–75	55–65	<55	–
Renal function	Urea (mmol l^−1^)	<7.5	7.5–21	>21	–	–
Liver function	AST/ALT ratio	<1.7	1.7–2.5	>2.5	–	–
Metabolism	Lactate (mmol l^−1^)	<1.64	1.64–3	3–4	4–5	>5

*ROFA, Rat Organ Failure Assessment; MAP, mean arterial pressure; ALT, alanine aminotransferase; AST, aspartate aminotransferase*.

### Microcirculatory Measurements

The Incident Dark Field (IDF) imaging technique (CytoCam Video Microscope System; Braedius Medical, Huizen, the Netherlands) was used for non-invasive evaluation of the serosal microcirculation of the ileum. IDF imaging is optimized for visualization of hemoglobin-containing structures by illuminating the organ surface with linearly polarized light, where the filtered light reflected from the tissues is detected by a computer-controlled sensor (23). Images from an ileum segment were recorded in six, 50-frame-long, high-quality video clips (spatial resolution 14 megapixels; temporal resolution 60 fps). The video was recorded at separate locations of the terminal ileum by the same investigator. The records were saved as digital AVI-DV files to a hard drive and analyzed with an off-line software-assisted system (AVA 3.0, Automated Vascular Analysis, Academic Medical Center, University of Amsterdam). The screens recorded with the IDF imaging technique were divided into four equal quadrants, as recommended. The proportion of perfused vessels (PPV) was defined as the ratio of the perfused vessel lengths to total vessel lengths. The PPV values were calculated in all quadrants, and the software (Automated Vascular Analysis 3.0) for the device provided four individual PPV values, Q1 PPV, Q2 PPV, Q3 PPV, and Q4 PPV, respectively. The average of these individual values (Q1PPV–Q4PPV) is shown as % PPV in the illustrations.

The median PPV values for the four quadrants were used as a reference (median values for Q1–4) in calculating microvascular heterogeneity (MVH). Heterogeneity was defined as the average difference of the PPV values (%) between each quadrant and the reference value (the differences are given in absolute values) for each record using the following formula:

$$\begin{array}{l} MVH=\;[|(M_{\sum QPPV}-Q1PPV)|\\ \;+|(M_{\sum QPPV}-\;Q2PPV)|+|(M_{\sum QPPV}-Q3PPV)|\\ \;+|(M_{\sum QPPV}-\;Q4PPV)|]/\text{number of quadrants} \end{array}$$

**M** = median

**M**_∑QPPV_ = median of the total PPV in the four quadrants

**QxPPV** = PPV value for each individual quadrant.

This calculation provides numerical values for perfusion heterogeneity (24, 25).

### Measurement of Mitochondrial Respiration and Membrane Potential

Mitochondrial O_2_ consumption and mitochondrial membrane potential (ΔΨmt) were assessed from liver homogenates using High-Resolution FluoRespirometry (Oxygraph-2k; Oroboros Instruments, Innsbruck, Austria). Measurements were performed in a Mir05 respiration medium under continuous magnetic stirring at 37°C. Changes in ΔΨmt were assessed with safranin dye using Blue Fluorescence Sensor (Sigma Aldrich, St. Louis, MO, USA). DatLab software (Oroboros Instruments, Innsbruck, Austria) was employed for online display, respirometry data acquisition, and analysis. A detailed description of the FluoRespirometry protocol used can be found in the Supplementary Data 1.

### Statistical Analysis

Data analysis was performed with a statistical software package (SigmaStat for Windows; Jandel Scientific, Erkrath, Germany). Normality of data distribution was analyzed with the Shapiro–Wilk test. The Friedman analysis of variance on ranks was applied within groups. Time-dependent differences from the baseline for each group were assessed with Dunn's method. In this study, differences between groups were analyzed with the Kruskal–Wallis one-way analysis of variance on ranks, followed by Dunn's method. Median values and 75th and 25th percentiles are provided in the figures; *P* < 0.05 were considered significant.

## Results

### Hemodynamics and Oxygen Dynamics

**Figure 2:**
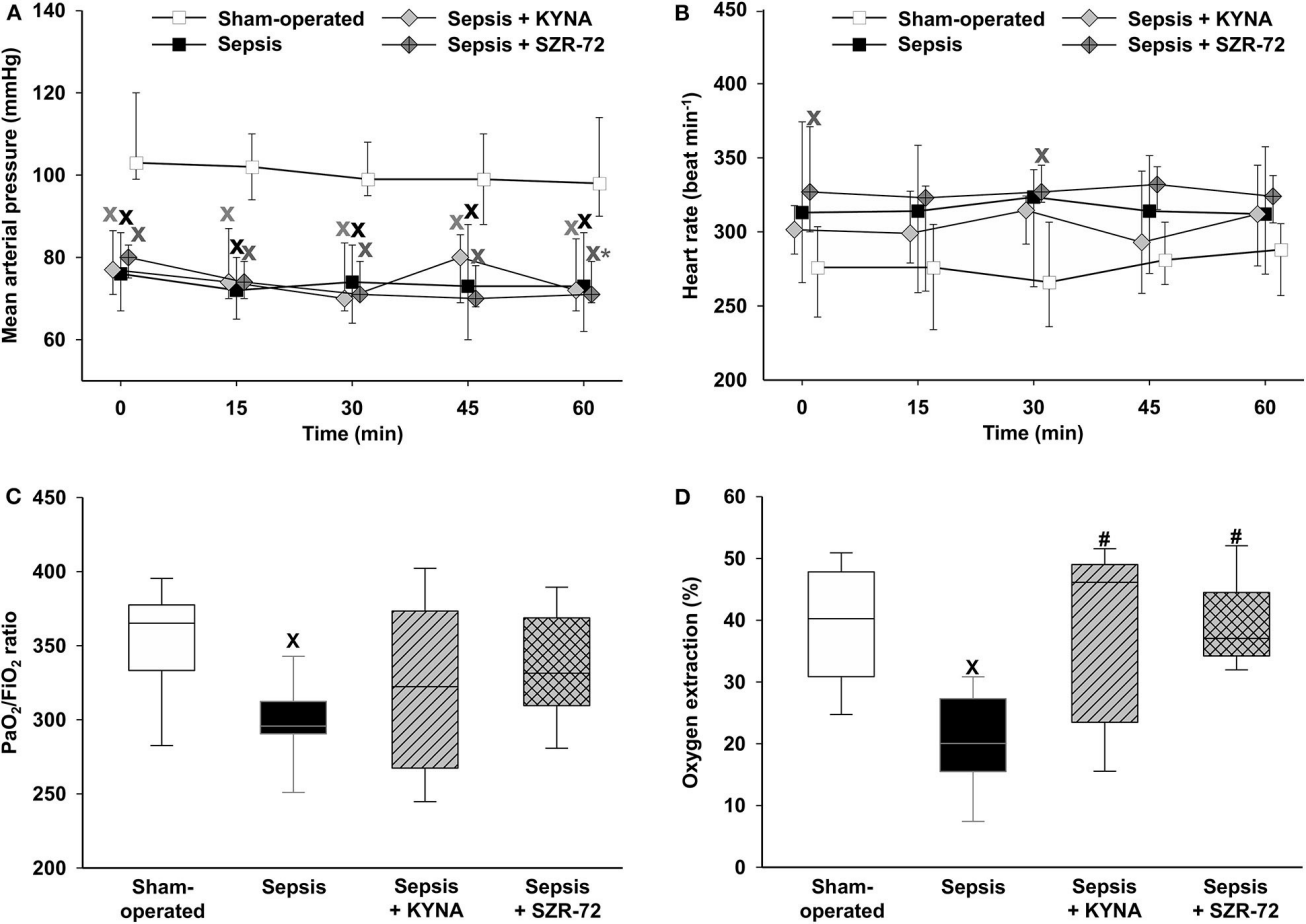
Time-dependent changes in mean arterial pressure **(A)**, heart rate **(B)**, PaO_2_/FiO_2_ ratio **(C)**, and oxygen extraction **(D)** in the sham-operated group (empty box) and in the different sepsis groups treated with the saline vehicle (black box), KYNA (gray diamond/striped gray box), and SZR-72 (dark gray diamond with cross/checked gray box). The plots demonstrate the median values (horizontal line in box plots) and the 25th (lower whisker) and 75th (upper whisker) percentiles. ^x^*P* < 0.05 vs. sham-operated; ^#^*P* < 0.05 vs. vehicle-treated sepsis; *P < 0.05 vs. 0 min.

Sepsis resulted in significant hypotension during the observation period, which was not altered by the treatments (Figure 2A). HR increased significantly in two time points (the 0th and 30th min of the monitoring period) in the SZR-72-treated group (Figure 2B). As compared with sham-operated animals, a decreased PaO_2_/FiO_2_ ratio was observed in the vehicle-treated sepsis group, whereas no significant changes were found in the other groups (Figure 2C). Sepsis reduced O_2_ER values as compared with the sham-operated group, whereas both KYNA and SZR-72 resulted in a significant improvement in this parameter (Figure 2D).

### Changes in Metabolic and Organ Dysfunction Markers

**Figure 3:**
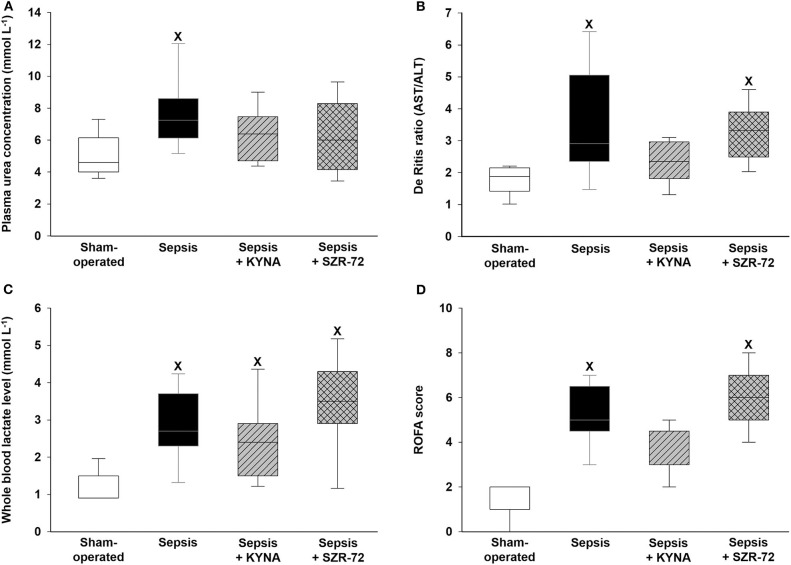
Changes in plasma urea **(A)**, De Ritis ratio (AST/ALT) **(B)**, whole blood lactate levels **(C)**, and ROFA score values **(D)** in the sham-operated group (empty box) and in the different sepsis groups treated with the saline vehicle (black box), KYNA (striped gray box), and SZR-72 (checked gray box). The plots demonstrate the median (horizontal line in the box) and the 25th (lower whisker) and 75th (upper whisker) percentiles. Between groups: Kruskal–Wallis test and Dunn's *post-hoc* test. ^x^*P* < 0.05 vs. sham-operated.

In the vehicle-treated sepsis group, plasma urea levels significantly increased, but urea levels in both treated groups were similar to those seen in the sham animals (Figure 3A). Hepatic cellular damage as indicated by the De Ritis ratio was evident in the vehicle-treated and SZR-72-treated sepsis groups, whereas this ratio did not differ between the sham-operated and KYNA-treated animals (Figure 3B). When ALT and AST values were evaluated separately (Supplementary Figures 1A,B), these changes were not influenced by the treatments. In comparison with the sham-operated group, all of the groups challenged with sepsis showed a similar extent of elevation in blood lactate levels (Figure 3C). The ROFA score was significantly higher in the vehicle-treated and SZR-72-treated sepsis groups than in the sham-operated group. The ROFA values in the KYNA-treated group were not significantly different from those in the septic group (Figure 3D).

### Changes in Inflammatory and Oxidative/Nitrosative Stress Markers

**Figure 4:**
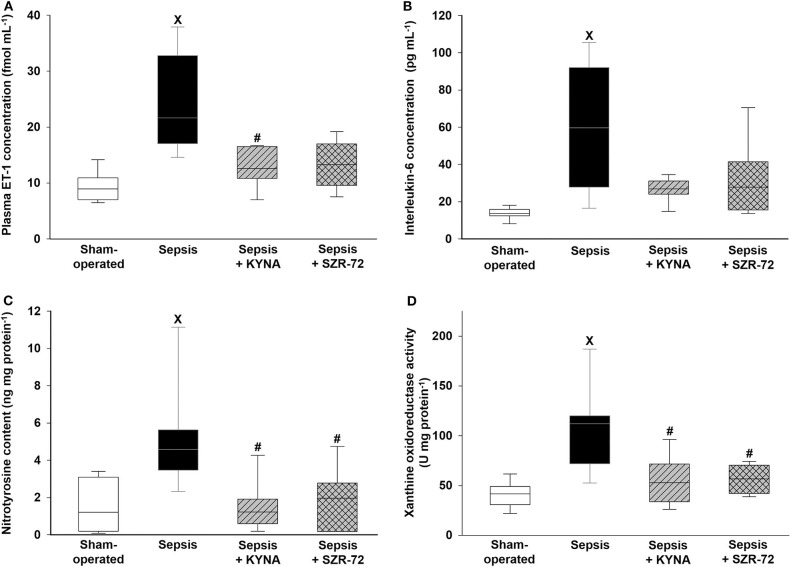
Changes in plasma endothelin-1 (ET-1) **(A)**, interleukin-6 (IL-6) **(B)**, ileal nitrotyrosine levels **(C)**, and xanthine oxidoreductase (XOR) activity **(D)** in the sham-operated group (empty box) and in the different sepsis groups treated with saline (black box), KYNA (striped gray box), and SZR-72 (checked gray box). The plots demonstrate the median (horizontal line in the box) and the 25th (lower whisker) and 75th (upper whisker) percentiles. Between groups: Kruskal–Wallis test and Dunn's *post-hoc* test. ^x^*P* < 0.05 vs. sham-operated; ^#^*P* < 0.05 vs. vehicle-treated sepsis.

Sepsis led to significant elevations in ET-1, IL-6, nitrotyrosine levels, and XOR activity (Figures 4A–D). All of these parameters remained, however, at the levels seen in the sham group in both the sepsis + KYNA and sepsis + SZR-72 groups.

### Microcirculatory Changes

**Figure 5:**
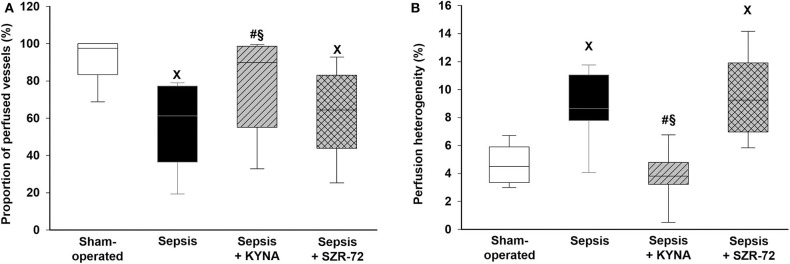
Changes in the proportion of perfused vessels **(A)** and in perfusion heterogeneity values **(B)** in the serosal layer of the ileum in the sham-operated group (empty box) and in the different sepsis groups treated with saline (black box), KYNA (striped gray box), and SZR-72 (checked gray box). The plots demonstrate the median (horizontal line in the box) and the 25th (lower whisker) and 75th (upper whisker) percentiles. Between groups: Kruskal–Wallis test and Dunn's *post-hoc* test. ^x^*P* < 0.05 vs. sham-operated; ^#^*P* < 0.05 vs. vehicle-treated sepsis; ^§^*P* < 0.05 sepsis + KYNA vs. sepsis + SZR-72.

Sepsis-induced microcirculatory perfusion disorders manifested in lower levels of capillary perfusion and increased perfusion heterogeneity as compared with those in the sham group (Figures 5A,B). The values of these parameters did not differ between the sepsis and sepsis + SZR-72 groups and between the sham and sepsis + KYNA groups. KYNA was significantly more effective in ameliorating sepsis-related changes than SZR-72.

### Changes in Mitochondrial Respiration

**Figure 6:**
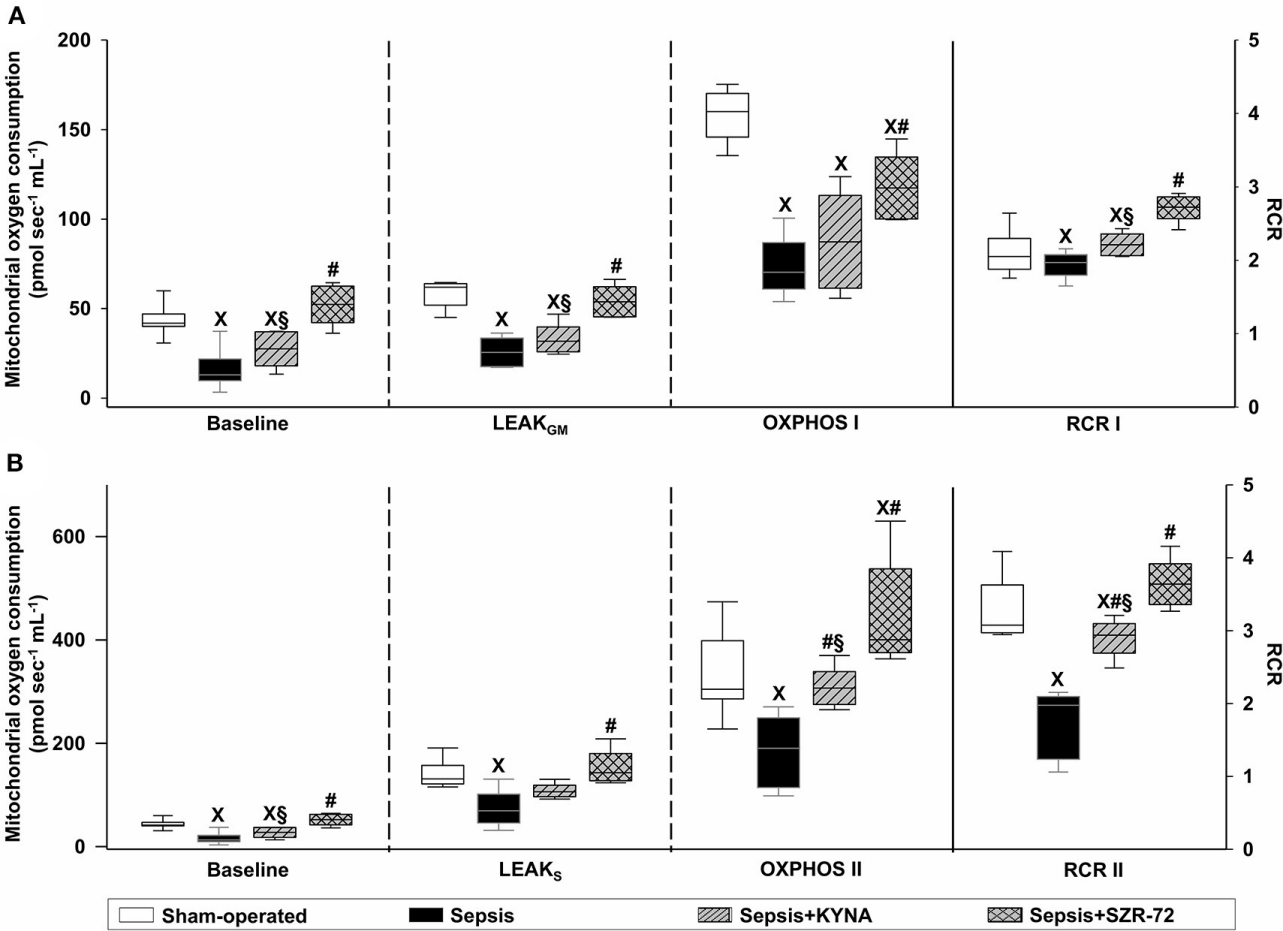
Baseline respiration, substrate oxidation (LEAK_GM_ and LEAK_S_), complex I- **(A)** and complex II-linked **(B)** OXPHOS capacities (OXPHOS I and OXPHOS II; left axis), and respiratory acceptor control ratios (CI, CII RCR; right axis) in the sham-operated group (empty box) and in the different sepsis groups treated with the saline vehicle (black box), KYNA (striped gray box), and SZR-72 (checked gray box). The plots illustrate the median (horizontal line in the box) and the 25th (lower whisker) and 75th (upper whisker) percentiles. ^X^*P* < 0.05 vs. sham-operated; ^#^*P* < 0.05 vs. vehicle-treated sepsis; ^§^*P* < 0.05 sepsis + KYNA vs. sepsis + SZR-72.

Baseline respiration without external substrate (BLresp) and respiration following the oxidation of complex I- and complex II-linked substrates (LEAK_GM_ and LEAK_S_) were significantly decreased in sepsis (Figures 6A,B). KYNA administration did not modify sepsis-induced changes in BLresp, LEAK_GM_, and LEAK_S_. In this respect, treatment with SZR-72 preserved mitochondrial respiration with and without NADH- and FADH_2_-linked substrates (Figures 6A,B). In addition, sepsis significantly decreased complex I- and complex II-linked OXPHOS. Both KYNA and SZR-72 increased complex II-linked OXPHOS capacity, whereas SZR-72 was able to restore complex I-linked OXPHOS completely (Figure 6A). As a result of septic insult, respiratory acceptor control ratios (RCR I and RCR II) were markedly decreased. These parameters were significantly improved by KYNA and completely reversed by SZR-72 treatment (Figures 6A,B).

### Changes in Mitochondrial Membrane Potential (ΔΨmt)

**Figure 7:**
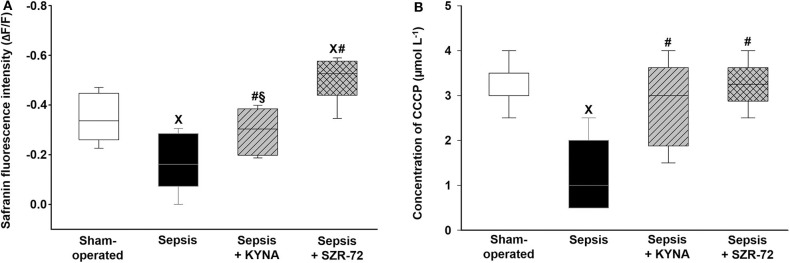
Succinate-induced hyperpolarization **(A)** and CCCP-induced loss of mitochondrial membrane potential **(B)** in the sham-operated group (empty box) and in the different sepsis groups treated with the saline vehicle (black box), KYNA (striped box), and SZR-72 (checked box). The plots illustrate the median (horizontal line in the box) and the 25th (lower whisker) and 75th (upper whisker) percentiles. ^x^*P* < 0.05 vs. sham-operated; ^#^*P* < 0.05 vs. vehicle-treated sepsis; ^§^*P* < 0.05 sepsis + KYNA vs. sepsis + SZR-72.

The addition of complex II substrate resulted in a sharp decrease in fluorescence signal (F_LEAK_) within 90 s, indicating an increase of ΔΨmt (succinate-induced hyperpolarization) (Figure 7A). As a result of the septic insult, there was a comparatively lower decrease in safranin fluorescence after succinate (Figure 7B), reflecting a significant decrease in ΔΨmt. Treatment with KYNA markedly improved, whereas SZR-72 completely restored the sepsis-induced decrease in ΔΨmt. Stimulation with CCCP resulted in depolarization and collapse of ΔΨmt at a critical uncoupler concentration. These changes were characterized as (I) stepwise increases in safranin fluorescence (depolarization) and (II) stabilization of fluorescence signal (loss of ΔΨmt).

In comparison with the sham-operated group, CCCP-mediated loss of ΔΨmt was obtained at lower uncoupler concentration in the vehicle-treated septic group (Figure 7B). KYNA and SZR-72 therapies significantly increased the concentration of CCCP used for ΔΨmt disruption. Among these treatments, SZR-72 had a more profound effect on ΔΨmt preservation (Figures 7A,B).

## Discussion

The present study demonstrates the distinct effects of KYNA and its synthetic analog on microcirculation and mitochondrial function in experimental sepsis. Proper analgesia, fluid resuscitation, and assessment of organ failure were conducted according to the standards in the Minimum Quality Threshold in Pre-clinical Sepsis Studies (MQTiPSS) guidelines (19). The clinical relevance of this model was confirmed by the presence of hypotension, impaired pulmonary function and oxygen extraction, and elevations in lactate levels and in oxidative/nitrosative stress as well as microcirculatory and mitochondrial dysfunctions. Sepsis-induced organ injuries were characterized by a newly developed, rat-specific organ failure (ROFA) scoring system based on threshold values adopted from the relevant literature (21). The scoring system included the following: (I–II) cardiovascular and respiratory dysfunctions were determined by MAP and PaO_2_/FiO_2_ values, respectively. (In MAP scoring, anesthesia-related effects were also taken into account, whereas PaO_2_/FiO_2_ was used in accordance with clinical practice.) (III) The AST/ALT ratio was considered to assess hepatocellular damage based on the observation that AST depicts liver-related injury more specifically than ALT (22). (IV) Renal damage was characterized by plasma urea changes (26) based on threshold values suggested by Zhai et al. (21). (V) The ROFA score was supplemented with consideration of lactate values (indicative of cellular hypoxia) according to Zhai et al. (21). This novel, rat-specific scoring system was applicable to trace development of MOF in this rat sepsis model and indicated differences between the efficacy of KYNA and SZR-72. Both treatments reduced lung and kidney dysfunctions to a similar extent due to the decreased level of inflammatory markers and their enhanced antioxidant/anti-nitrosative effect. However, only KYNA treatment reduced hypoxia-sensitive ET-1 levels, also displaying a tendency toward amelioration of liver injury and ROFA score values.

Deteriorating tissue perfusion is a key element in sepsis pathophysiology and in the development of MOF (4). We observed that both KYNA and SZR-72 prevented a sepsis-induced decrease in oxygen extraction to the same extent, but only KYNA ameliorated sepsis-related microcirculatory perfusion deficit (reduction in capillary perfusion and an increase in perfusion heterogeneity) significantly. This distinct effect of compounds is possibly due to their different structure- and receptor-related characteristics (27).

The potential direct microcirculatory effects of KYNA in the ileum are unknown, but it has been shown to increase global and cortical renal blood flow (and to improve renal excretion) under physiological conditions and to reduce renal oxidative stress during ischemia–reperfusion injury (28, 29). Based on our results, some of these micro-hemodynamic effects can also be linked to a reduced ET-1 release elicited by KYNA.

NMDA receptor-related microcirculation improvement can be explained by many mechanisms. Firstly, antagonism of NMDA receptors expressed on the surface of smooth muscle cells brings about reduced intracellular Ca^2+^ levels (30), resulting in smooth muscle relaxation (31). On the other hand, a reduction of the levels of pro-inflammatory IL-6, XOR activity, and ROS-sensitive vasoconstrictor ET-1 release by KYNA may attenuate the cytokine- and ROS-induced vasoconstriction of microvessels. Although the use of vasodilator therapy in sepsis is debated (6, 32, 33), a reduction of circulating ET-1 levels through a combined ET_A_/ET_B_ receptor-targeted treatment regimen has been demonstrated to ameliorate microcirculatory deficit in sepsis (17).

Further, KYNA may also act as an agonist for the orphan receptor GPR35 and reduce inflammation independently of the NMDA receptors. This receptor is expressed at high levels in intestine and immune cells (34), and the concentrations of KYNA required to induce effects differ between NMDA-R and GPR35. Compared with NMDA-R, lower concentrations of KYNA are able to elicit a response when binding to GPR35 (35). Taking into account that (1) KYNA is an endogenous ligand for GPR35 and that (2) only KYNA, but not the synthetic analog, was able to restore the perfusion disturbances in the ileum, a GPR35-mediated mechanism cannot be ruled out.

The KYNA- and its analog-based treatments also had different effects on mitochondrial function, with the ameliorating effects of SZR-72 being more pronounced. A considerable decrease in substrate- and ADP-stimulated respirations was accompanied by a decrease in RCR and ΔΨmt after sepsis insult (17). A decrease of ΔΨmt may originate in complex metabolic, functional, and membrane integrity changes within the organelle and may explain the decline in mitochondrial O_2_ consumption and lower interdependence (coupling) of ATP synthesis with ETS. Although an O_2_-independent glycolytic pathway for ATP production has been documented, this alternate route may not be effective for energy production in sepsis. Of note, ATP depletion is not a unique component of sepsis-induced mitochondrial dysfunction; organellar ROS production, elevated mitochondrial DNA level, transition pore opening-mediated apoptosis, and necrosis are fundamental mitochondrial events, which lead to cellular injury (36, 37). In addition, the release of mitochondrial components of damage-associated molecular patterns to the extracellular space may further aggravate an inflammatory response (38). Taken together, all these processes are intimately involved in the progression of sepsis and contribute to MOF.

The role of endogenous KYNA on mitochondrial physiology is still unmapped; however, exogenous KYNA was able to improve OXPHOS II and ΔΨmt without affecting OXPHOS I. This difference in complex activities may arise from the fact that complex I is more susceptible to cellular injury than complex II. Oxidation of glutamate requires pyridine nucleotides, and these with other cofactors may be lost during sepsis (39).

Our findings are in agreement with the study by Ferreira et al. (40), in which KYNA ameliorated several aspects of mitochondrial function in a neurodegeneration model. Similar to our results, KYNA administration significantly improved succinate dehydrogenase activity and ΔΨmt. In addition, KYNA preserved mitochondrial mass, enhanced antioxidant enzyme levels, and reduced ROS production after quinolinic acid-induced cell injury (40). In adipose tissue, KYNA modulated energy utilization (increased lipid metabolism and mitochondrial respiration) via a GPR35 pathway (41).

In our experiments, administration of SZR-72 markedly improved the key indices of mitochondrial function. Previous studies with the KYNA analog revealed a more pronounced anti-inflammatory response in animal models of colitis and neuro-inflammation than that seen with KYNA (10, 16, 42), but both KYNA and SZR-72 reduced oxidative/nitrosative stress marker levels [XOR, nitric oxide synthase (NOS), and myeloperoxidase (MPO) activities] (10) and attenuated glutamate expression (42). Compared with KYNA, the effects of SZR-72 were more pronounced in this sepsis model, and a remarkable increase in ADP-stimulated respirations (OXPHOS I and II), RCR, and ΔΨmt was found in liver homogenate after sepsis induction. Preservation of these parameters is fundamental for better O_2_ utilization (less ETS-linked ROS is generated), maintenance of ATP production, and provision of ΔΨmt for mitochondrial transport.

Several hypotheses may explain the mechanism behind the more advantageous mitochondrial effects of SZR-72. First, the physico-chemical properties of KYNA and SZR-72 are different, thus possibly influencing crossing through the BBB and membranes (16). There is evidence that KYNA only crosses the BBB poorly, whereas SZR-72 is BBB-permeable due to a water-soluble side chain with an extra cationic center (7, 43). Apart from BBB, a facilitated membrane crossing of SZR-72 may affect intracellular signaling, including the activation of antioxidative/anti-apoptotic pathways.

A second scenario is a distinct molecule binding at the NMDA-R glycine site. More recently, NMDA-Rs were shown to be present in the inner mitochondrial membrane (mtNMDA-R) (44), where they may play a regulative role in (1) Ca^2+^ transport, (2) ROS production, and (3) metabolic switching during hypoxia (45). Under these circumstances, it may well be that SZR-72 has a much higher affinity for either plasma membrane or mtNMDA-R than KYNA. In addition, a protein–protein interaction between NMDA-R and an ND2 subunit of complex I was found through an Src adapter protein (12). It cannot be ruled out that KYNA and SZR-72 influence this interaction and regulate mitochondrial homeostasis differently.

The influence of sepsis on NMDA-R expression and glutamate levels is incompletely characterized. We did not examine changes in NMDA-R subunits due to technical limitations, but other laboratories have found a marked increase in lung NR1 and NR2A contents in a cecal ligation and puncture (CLP) sepsis model. Furthermore, treatment with the NMDA-R antagonist MK-801 lowered lactate dehydrogenase and oxidative damage and improved survival 144 h after sepsis induction in rats (46). Similarly, survival was markedly increased (by 80%), and markers of inflammation were reduced in MK-801-treated mice 48 h after lipopolysaccharide (LPS) stimuli (47). In this model, LPS also raised the glutamate level in bronchoalveolar lavage fluid. Based on these findings and the fact that KYNA is a natural antagonist, a similar, NMDA-R-linked mechanism, at least in part, cannot be ruled out in our polymicrobial sepsis model.

Our study has limitations. Firstly, the observation timeline was relatively short, and therefore longer end points, such as mortality, with longer pharmacological effects should also be examined in follow-up studies. Our newly described ROFA score also seems to be a potentially useful tool for this purpose, but it will be necessary to validate the system under other experimental conditions as well. The effect of the ketamine-containing anesthesia on the results cannot be disregarded. Similarly, despite our intention to follow all of the recommendations in the MQTiPSS guidelines, antimicrobial therapy was excluded from the protocol due to the known influence on mitochondrial respiration (48).

In conclusion, both treatments with KYNA and its synthetic analog attenuated the deleterious consequences of oxidative/nitrosative stress and resulted in lower inflammatory mediator release. Administration of SZR-72 may directly regulate mitochondrial respiration and ATP synthesis, whereas treatment with KYNA primarily ameliorates microcirculatory dysfunction and consequently restores organelle function. Further experiments are surely needed to clarify the exact mechanism behind these compounds, but these results suggest that therapies with KYNA or its synthetic analog, SZR-72, against MMDS might be a supportive intervention in the treatment of sepsis.

## Data Availability Statement

The raw data supporting the conclusions of this article will be made available by the authors, without undue reservation.

## Ethics Statement

The animal study was reviewed and approved by National Competent Authority of Hungary.

## Author Contributions

LJ, AR, RF, ST, MP, and JK performed experiments and wrote the manuscript. ST and AS prepared figures. FF and IS contributed to new KYNA analog. AS, JK, MB, and LV supervised and edited the manuscript. All authors contributed to the article and approved the submitted version.

## Conflict of Interest

The authors declare that the research was conducted in the absence of any commercial or financial relationships that could be construed as a potential conflict of interest.

## References

[1] Singer M Deutschman CS Seymour CW Shankar-Hari M Annane D Bauer M The Third International Consensus Definitions for Sepsis and Septic Shock (Sepsis-3). JAMA. (2016) 315:801 10.1001/jama.2016.0287 26903338 PMC4968574

[2] De Backer D Orbegozo Cortes D Donadello K Vincent J-L. Pathophysiology of microcirculatory dysfunction and the pathogenesis of septic shock. Virulence. (2014) 5:73–79. 10.4161/viru.26482 24067428 PMC3916386

[3] Arulkumaran N Deutschman CS Pinsky MR Zuckerbraun B Schumacker PT Gomez H. Mitochondrial function in sepsis. Shock. (2016) 45:271–81. 10.1097/SHK.0000000000000463 26871665 PMC4755359

[4] Balestra GM Legrand M Ince C. Microcirculation and mitochondria in sepsis: getting out of breath. Curr Opin Anaesthesiol. (2009) 22:184–90. 10.1097/ACO.0b013e328328d31a 19307893

[5] Armstrong BA Betzold RD May AK. Sepsis and septic shock strategies. Surg Clin North Am. (2017) 97:1339–79. 10.1016/j.suc.2017.07.003 29132513

[6] Moore JPR Dyson A Singer M Fraser J. Microcirculatory dysfunction and resuscitation: why, when, and how. Br J Anaesth. (2015) 115:366–75. 10.1093/bja/aev163 26269467

[7] Vécsei L Szalárdy L Fülöp F Toldi J. Kynurenines in the CNS: recent advances and new questions. Nat Rev Drug Discov. (2013) 12:64–82. 10.1038/nrd3793 23237916

[8] Rameaut GA Chiu LY Ziff EB. Bidirectional regulation of neuronal nitric-oxide synthase phosphorylation at serine 847 by the N-methyl-D-aspartate receptor. J Biol Chem. (2004) 279:14307–14. 10.1074/jbc.M311103200 14722119

[9] Dabrowski W Kocki T Pilat J Parada-Turska J Malbrain MLNG. Changes in plasma kynurenic acid concentration in septic shock patients undergoing continuous veno-venous haemofiltration. Inflammation. (2014) 37:223–34. 10.1007/s10753-013-9733-9 24043287 PMC3929023

[10] Kaszaki J Érces D Varga G Szabó A Vécsei L Boros M. Kynurenines and intestinal neurotransmission: the role of N-methyl-d-aspartate receptors. J Neural Transm. (2012) 119:211–23. 10.1007/s00702-011-0658-x 21617892

[11] Hogan-Cann AD Anderson CM. Physiological roles of non-neuronal NMDA receptors. Trends Pharmacol Sci. (2016) 37:750–67. 10.1016/j.tips.2016.05.012 27338838

[12] Gingrich JR Pelkey KA Fam SR Huang Y Petralia RS Wenthold RJ. Unique domain anchoring of Src to synaptic NMDA receptors via the mitochondrial protein NADH dehydrogenase subunit 2. Proc Natl Acad Sci USA. (2004) 101:6237–42. 10.1073/pnas.0401413101 15069201 PMC395953

[13] Tanaka M Bohár Z Vécsei L. Are kynurenines accomplices or principal villains in dementia? Maintenance of kynurenine metabolism. Molecules. (2020) 25:564. 10.3390/molecules25030564 32012948 PMC7036975

[14] Walczak K Wnorowski A Turski WA Plech T. Kynurenic acid and cancer: facts and controversies. Cell Mol Life Sci. (2020) 77:1531–50. 10.1007/s00018-019-03332-w 31659416 PMC7162828

[15] Fülöp F Szatmári I Vámos E Zádori D Toldi J Vécsei L. Syntheses, transformations and pharmaceutical applications of kynurenic acid derivatives. Curr Med Chem. (2009) 16:4828–42. 10.2174/092986709789909602 19929784

[16] Knyihar-Csillik E Mihaly A Krisztin-Peva B Robotka H Szatmari I Fulop F. The kynurenate analog SZR-72 prevents the nitroglycerol-induced increase of c-fos immunoreactivity in the rat caudal trigeminal nucleus: comparative studies of the effects of SZR-72 and kynurenic acid. Neurosci Res. (2008) 61:429–32. 10.1016/j.neures.2008.04.009 18541319

[17] Rutai A Fejes R Juhász L Tallósy SP Poles MZ Földesi I. Endothelin A and B receptors. Shock. (2019) 54:87–95. 10.1097/SHK.0000000000001414 31318833

[18] Rademann P Weidinger A Drechsler S Meszaros A Zipperle J Jafarmadar M. Mitochondria-targeted antioxidants SkQ1 and MitoTEMPO failed to exert a long-term beneficial effect in murine polymicrobial sepsis. Oxid Med Cell Longev. (2017) 2017:6412682. 10.1155/2017/6412682 29104729 PMC5625755

[19] Osuchowski MF Ayala A Bahrami S Bauer M Boros M Cavaillon J-M. Minimum quality threshold in pre-clinical sepsis studies (MQTiPSS). Shock. (2018) 50:377–80. 10.1097/SHK.0000000000001212 30106875 PMC6133201

[20] Beckman JS Parks DA Pearson JD Marshall PA Freeman BA. A sensitive fluorometric assay for measuring xanthine dehydrogenase and oxidase in tissues. Free Radic Biol Med. (1989) 6:607–15. 10.1016/0891-5849(89)90068-3 2753392

[21] Zhai X Yang Z Zheng G Yu T Wang P Liu X. Lactate as a potential biomarker of sepsis in a rat cecal ligation and puncture model. Mediators Inflamm. (2018) 2018:8352727. 10.1155/2018/8352727 29706801 PMC5863333

[22] Botros M Sikaris KA. The De Ritis ratio: the test of time. Clin Biochem Rev. (2013) 34:117–30. 24353357 PMC3866949

[23] Aykut G Veenstra G Scorcella C Ince C Boerma C. Cytocam-IDF (incident dark field illumination) imaging for bedside monitoring of the microcirculation. Intens Care Med Exp. (2015) 3:40. 10.1186/s40635-015-0040-7 26215807 PMC4512989

[24] De Backer D Hollenberg S Boerma C Goedhart P Büchele G Ospina-Tascon G. How to evaluate the microcirculation: report of a round table conference. Crit Care. (2007) 11:R101. 10.1186/cc6118 17845716 PMC2556744

[25] Massey MJ Shapiro NI. A guide to human in vivo microcirculatory flow image analysis. Crit Care. (2016) 20:35. 10.1186/s13054-016-1213-9 26861691 PMC4748457

[26] Wang K Xie S Xiao K Yan P He W Xie L Biomarkers of sepsis-induced acute kidney injury. Biomed Res Int. (2018) 2018:6937947 10.1155/2018/6937947 29854781 PMC5941779

[27] Mándi Y Endrész V Mosolygó T Burián K Lantos I Fülöp F The opposite effects of kynurenic acid and different kynurenic acid analogs on tumor necrosis factor-α (TNF-α) production and tumor necrosis factor-stimulated gene-6 (TSG-6) expression. Front Immunol. (2019) 10:1406 10.3389/fimmu.2019.01406 31316502 PMC6611419

[28] Badzyńska B Zakrocka I Sadowski J Turski WA Kompanowska-Jezierska E. Effects of systemic administration of kynurenic acid and glycine on renal haemodynamics and excretion in normotensive and spontaneously hypertensive rats. Eur J Pharmacol. (2014) 743:37–41. 10.1016/j.ejphar.2014.09.020 25263305

[29] Pundir M Arora S Kaur T Singh R Singh AP. Effect of modulating the allosteric sites of N-methyl-D-aspartate receptors in ischemia-reperfusion induced acute kidney injury. J Surg Res. (2013) 183:668–77. 10.1016/j.jss.2013.01.040 23498342

[30] Wirthgen E Hoeflich A Rebl A Günther J. Kynurenic Acid: the Janus-faced role of an immunomodulatory tryptophan metabolite and its link to pathological conditions. Front Immunol. (2018) 8:1957. 10.3389/fimmu.2017.01957 29379504 PMC5770815

[31] Adelstein RS Sellers JR. Effects of calcium on vascular smooth muscle contraction. Am J Cardiol. (1987) 59:4–10. 10.1016/0002-9149(87)90076-2 3028118

[32] Trzeciak S Glaspey LJ Dellinger RP Durflinger P Anderson K Dezfulian C. Randomized controlled trial of inhaled nitric oxide for the treatment of microcirculatory dysfunction in patients with sepsis^*^. Crit Care Med. (2014) 42:2482–92. 10.1097/CCM.0000000000000549 25080051

[33] Boerma EC Koopmans M Konijn A Kaiferova K Bakker AJ van Roon EN. Effects of nitroglycerin on sublingual microcirculatory blood flow in patients with severe sepsis/septic shock after a strict resuscitation protocol: a double-blind randomized placebo controlled trial. Crit Care Med. (2010) 38:93–100. 10.1097/CCM.0b013e3181b02fc1 19730258

[34] Wang J Simonavicius N Wu X Swaminath G Reagan J Tian H. Kynurenic acid as a ligand for orphan G protein-coupled receptor GPR35. J Biol Chem. (2006) 281:22021–8. 10.1074/jbc.M603503200 16754668

[35] Turski MP Turska M Paluszkiewicz P Parada-Turska J Oxenkrug GF. Kynurenic Acid in the digestive system-new facts, new challenges. Int J Tryptophan Res. (2013) 6:47–55. 10.4137/IJTR.S12536 24049450 PMC3772988

[36] Bantel H Schulze-Osthoff K. Cell death in sepsis: a matter of how, when, and where. Crit Care. (2009) 13:173. 10.1186/cc7966 19678906 PMC2750164

[37] Harrington JS Choi AMK Nakahira K. Mitochondrial DNA in sepsis. Curr Opin Crit Care. (2017) 23:284–90. 10.1097/MCC.0000000000000427 28562385 PMC5675027

[38] Nakahira K Hisata S Choi AMK The roles of mitochondrial damage-associated molecular patterns in diseases. Antioxidants Redox Signal. (2015) 23:1329–50. 10.1089/ars.2015.6407 PMC4685486 26067258

[39] Hart DW Gore DC Rinehart AJ Asimakis GK Chinkes DL. Sepsis-induced failure of hepatic energy metabolism. J Surg Res. (2003) 115:139–47. 10.1016/S0022-4804(03)00284-1 14572785

[40] Ferreira FS Biasibetti-Brendler H Pierozan P Schmitz F Bertó CG Prezzi CA. Kynurenic acid restores Nrf2 levels and prevents quinolinic acid-induced toxicity in rat striatal slices. Mol Neurobiol. (2018) 55:8538–49. 10.1007/s12035-018-1003-2 29564809

[41] Agudelo LZ Ferreira DMS Cervenka I Bryzgalova G Dadvar S Jannig PR. Kynurenic acid and Gpr35 regulate adipose tissue energy homeostasis and inflammation. Cell Metab. (2018) 27:378–92. 10.1016/j.cmet.2018.01.004 29414686

[42] Lukács M Warfvinge K Tajti J Fülöp F Toldi J Vécsei L. Topical dura mater application of CFA induces enhanced expression of c-fos and glutamate in rat trigeminal nucleus caudalis: attenuated by KYNA derivate (SZR72). J Headache Pain. (2017) 18:39. 10.1186/s10194-017-0746-x 28337634 PMC5364126

[43] Fülöp F Szatmári I Toldi J Vécsei L. Modifications on the carboxylic function of kynurenic acid. J Neural Transm. (2012) 119:109–14. 10.1007/s00702-011-0721-7 21997444

[44] Nesterov SV Skorobogatova YA Panteleeva AA Pavlik LL Mikheeva IB Yaguzhinsky LS. NMDA and GABA receptor presence in rat heart mitochondria. Chem Biol Interact. (2018) 291:40–6. 10.1016/j.cbi.2018.06.004 29883723

[45] Selin AA Lobysheva NV Nesterov SV Skorobogatova YA Byvshev IM Pavlik LL. On the regulative role of the glutamate receptor in mitochondria. Biol Chem. (2016) 397:445–58. 10.1515/hsz-2015-0289 26812870

[46] da Cunha AA Pauli V Saciura VC Pires MG Constantino LC de Souza B. N-methyl-D-aspartate glutamate receptor blockade attenuates lung injury associated with experimental sepsis. Chest. (2010) 137:297–302. 10.1378/chest.09-1570 19837828

[47] Zhe Z Hongyuan B Wenjuan Q Peng W Xiaowei L Yan G. Blockade of glutamate receptor ameliorates lipopolysaccharide-induced sepsis through regulation of neuropeptides. Biosci Rep. (2018) 38:BSR20171629. 10.1042/BSR20171629 29440461 PMC5938426

[48] Moullan N Mouchiroud L Wang X Ryu D Williams EG Mottis A. Tetracyclines disturb mitochondrial function across eukaryotic models: a call for caution in biomedical research. Cell Rep. (2015) 10:1681–91. 10.1016/j.celrep.2015.02.034 25772356 PMC4565776

