# Supplementary material for: Divergent Effects of the N-Methyl-D-Aspartate Receptor Antagonist Kynurenic Acid and the Synthetic Analog SZR-72 on Microcirculatory and Mitochondrial Dysfunction in Experimental Sepsis
Source: Front Med (Lausanne). 2020 Nov 27;7:566582. doi: 10.3389/fmed.2020.566582 (PMC7729001; doi:10.3389/fmed.2020.566582)
Supplement: Supplementary file 1 [file Data_Sheet_1.pdf]

Supplementary Data 1

Assessment of mitochondrial respiration

Mitochondrial O_2 consumption (JVO_2; volume-specific O_2 flux) was measured in liver homogenates using High-Resolution FluoRespirometry (Oxygraph-2k, Oroboros Instruments, Innsbruck, Austria). Briefly, 200–300 mg tissue samples obtained from the left lateral lobe of the liver were cut into smaller pieces, washed in phosphate-buffered saline five times and then homogenized with a Potter–Elvehjem tissue grinder in an isolation medium containing 250 mmol L^{-1} sucrose, 0.5 mmol L^{-1} Na_2EDTA, 10 mmol L^{-1} Tris and 1 g L^{-1} bovine serum albumin. Calibration and measurements were performed with continuous stirring (750 rpm) at 37°C in a 2 mL Mir05 respiration medium (1,2). Chamber O_2 concentration was maintained between 200 and 50 µmol L^{-1} during the experiments. After stabilization of respiration, NADH- and $FADH_2$-supported LEAK respiration and complex I- and II-linked maximal capacities of oxidative phosphorylation (OXPHOS I and OXPHOS II; Supplementary Data 1 Figure A–B) were determined in the presence of substrates ($LEAK_{GM}$; 10 mmol L^{-1} glutamate, 2 mmol L^{-1} malate; and $LEAK_S$; 10 mmol L^{-1} succinate) and saturating concentration of ADP (2.5 mmol L^{-1}). Rotenone (0.5 µmol L^{-1}; complex I inhibitor) was only administered prior to succinate to (a) block ROS generation via reverse electron transport and (b) prevent accumulation of oxaloacetate, a known endogenous inhibitor of complex II. Following stimulation of OXPHOS, the integrity of the outer mitochondrial membrane was tested with exogenous cytochrome c (10 µmol L^{-1}). ATP synthase was inhibited by oligomycin (2.5 µmol L^{-1}) to assess LEAK respiration in a non-phosphorylating state ($LEAK_{Omy}$). Respiratory control ratio (RCR), an index of coupling between respiration and phosphorylation, was expressed as a ratio of OXPHOS to the $LEAK_{Omy}$ state. The electron transport system-independent respiration (or residual oxygen consumption; ROX) was evaluated following complex III inhibition with antimycin A (2.5 µmol L^{-1}). The DatLab software (Oroboros Instruments, Innsbruck, Austria) was used for online display, respirometry data acquisition and analysis.

Determination of changes of the mitochondrial membrane potential

Changes in mitochondrial membrane potential ($\Delta\Psi mt$) were assessed in liver homogenate with a cationic fluorescent probe, safranin (Sigma Aldrich, St. Louis, Mo., USA). The probe accumulates in energized mitochondria according to the inside negative potential with a concomitant change in absorption and fluorescence (3). A Blue Fluorescence Sensor (excitation 465 nm; gain for sensor: 1000; polarization voltage: 500 mV) connected to the windows on the glass chambers and the oxygraph was used to measure safranin fluorescence according to the manufacturer's instructions (https://wiki.oroboros.at/images/5/52/MiPNet20.13_Safranin_mt-membranepotential.pdf). The dye was dissolved in distilled water (1 mmol L^{-1} stock solution), stored in dark vials and titrated into the 2 mL O2k chamber up to 2 µmol L^{-1} of safranin final concentration. Previous data have shown that this tracer concentration does not affect complex II-linked coupled respiration (3) and fluorescence spectral changes are linearly related to $\Delta\Psi mt$ within a 0.5–2 µmol L^{-1} concentration range. The protocol for homogenate preparation was identical with those described earlier (1,2). All the measurements were performed in a Mir05 respiration medium under continuous magnetic stirring (750 rpm) at 37ºC. Stoppers were covered with black cover slips to prevent light penetrating the capillary and to avoid disruption of the fluorescence signal. Liver homogenates were energized with succinate (10 mmol L^{-1}) after inhibition complex I with rotenone (0.5 µmol L^{-1}). After stabilization respiration and fluorescence, a protonophorous uncoupler, carbonyl cyanide m-chlorophenyl hydrazone (CCCP), was titrated into a respiration chamber (0.5 µmol L^{-1} in steps) for the stepwise depolarization of $\Delta\Psi mt$. During CCCP stimulation, O_2 concentration was maintained above 50 µmol L^{-1} to induce maximum O_2 flux and to avoid the inhibitory effect of hypoxia on electron

transfer-pathway capacity. When $\Delta\Psi$mt was collapsed after optimum concentration of CCCP, uncoupled respiration was inhibited with antimycin A (2.5 μmol L^{-1}; ROX). Safranin fluorescence (F) was expressed as the rate of change in fluorescent signal and average resting fluorescence using the following formula: safranin fluorescence intensity $= \Delta F/F = (F_{LEAK} - F_{rest})/F_{rest}$, where F_{LEAK} is the indicator of fluorescence after succinate and F_{rest} is the average fluorescence signal before the addition of substrate (Supplementary Data 1 Figure C).

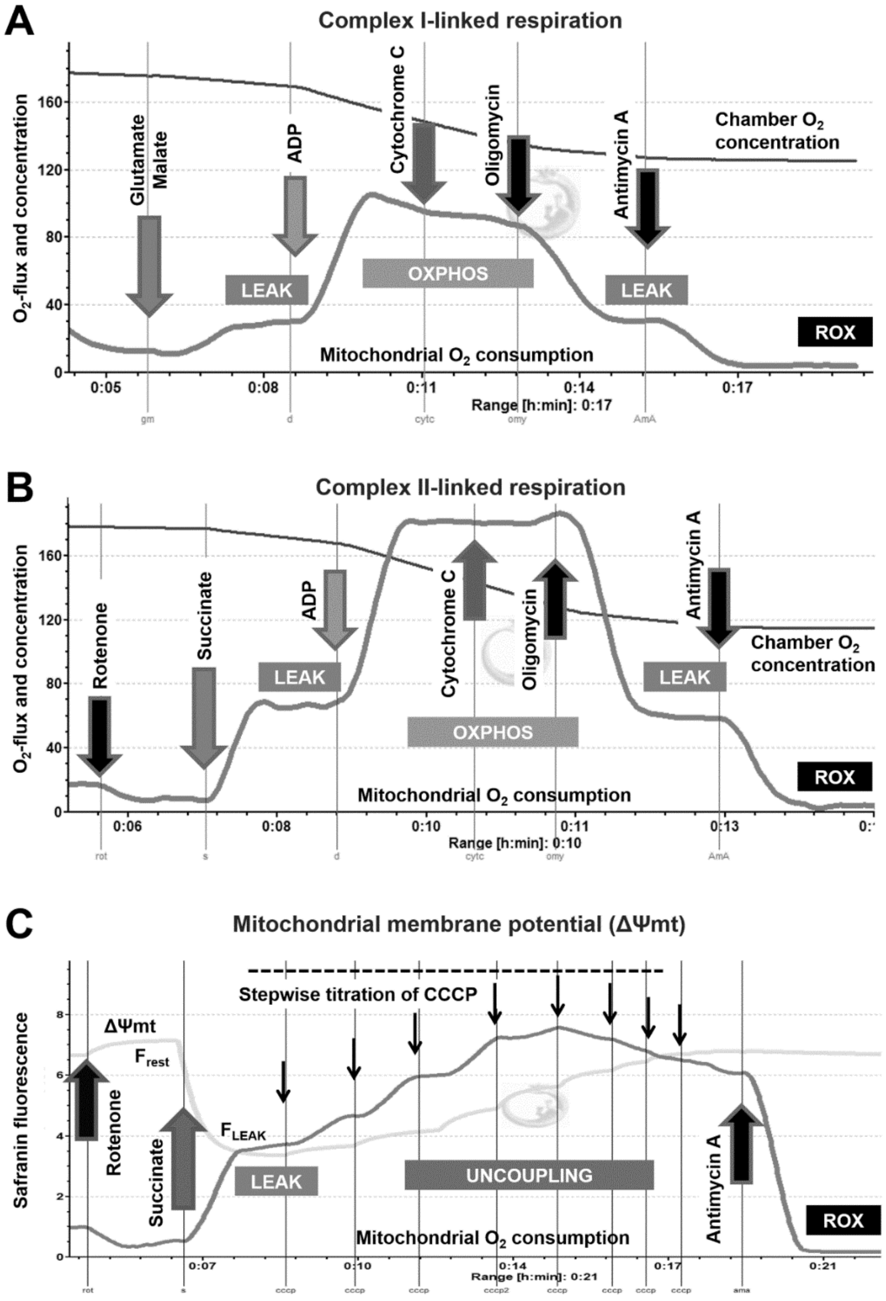

Supplementary Data 1 Figure. FluoRespirometric protocols. Complex I- and II-linked respiration, oxidative phosphorylation (OXPHOS) capacities (A,B) and mitochondrial membrane potential ($\Delta\Psi$mt; C) were assessed in energized liver homogenates. Mitochondrial electron transport system-dependent O_2 consumption was confirmed by blocking complex III with antimycin A (ROX; residual oxygen consumption). Substrate- and uncoupler-induced changes in $\Delta\Psi$mt were monitored in the presence of safranin fluorescent dye.

REFERENCES

1.Rutai A, Fejes R, Juhász L, Tallósy SP, Poles MZ, Földesi I, Mészáros AT, Szabó A, Boros M, Kaszaki J. Endothelin A and B Receptors. Shock (2019) Online ahead of print. doi:10.1097/SHK.0000000000001414

2.Nászai A, Terhes E, Kaszaki J, Boros M, Juhász L. Ca(2+)N It Be Measured? Detection of Extramitochondrial Calcium Movement With High-Resolution FluoRespirometry. Sci Rep (2019) 9:19229 doi:10.1038/s41598-019-55618-5

3.Krumschnabel G, Eigentler A, Fasching M, Gnaiger E. Use of safranin for the assessment of mitochondrial membrane potential by high-resolution respirometry and fluorometry. Methods Enzymol (2014) 542:163–81. doi:10.1016/B978-0-12-416618-9.00009-1
