# Supplementary material for: Divergent Effects of the N-Methyl-D-Aspartate Receptor Antagonist Kynurenic Acid and the Synthetic Analog SZR-72 on Microcirculatory and Mitochondrial Dysfunction in Experimental Sepsis
Source: Front Med (Lausanne). 2020 Nov 27;7:566582. doi: 10.3389/fmed.2020.566582 (PMC7729001; doi:10.3389/fmed.2020.566582)
Supplement: Supplementary file 2 [file Image_1.pdf]

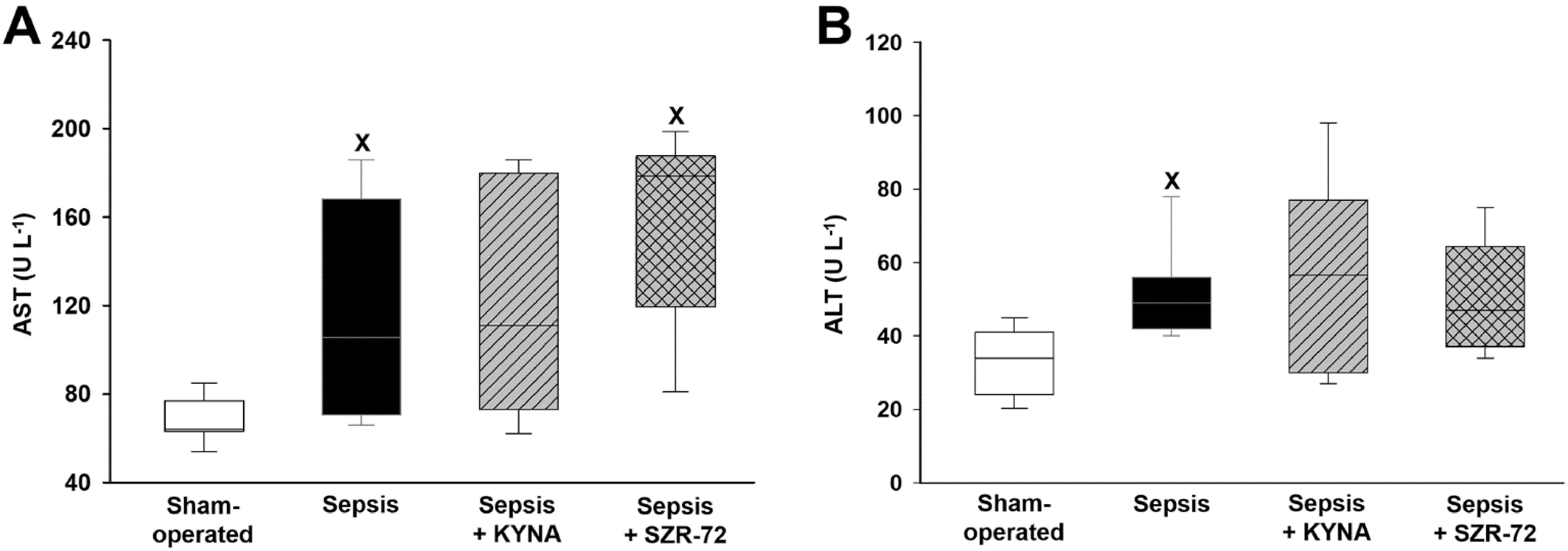

Figure S1. The plasma aspartate transaminase (AST) (A) and plasma alanine aminotransferase (ALT) (B) values in the sham-operated group (empty box) and in the different sepsis groups treated with the saline vehicle (black box), KYNA (striped gray box) and SZR-72 (checked gray box). The plots demonstrate the median (horizontal line in the box) and the 25th (lower whisker) and 75th (upper whisker) percentiles. Between groups: Kruskal–Wallis test and Dunn's post-hoc test. x $P < 0.05$ vs. sham-operated.
